# T-Wave Oversensing with Contemporary Implantable Cardioverter–Defibrillators

**DOI:** 10.3390/jcdd10100430

**Published:** 2023-10-17

**Authors:** Marc Strik, Sylvain Ploux, Romain Eschalier, Pierre Mondoly, Leslie Fontagne, F. Daniel Ramirez, Michel Haïssaguerre, Pierre Bordachar

**Affiliations:** 1 Bordeaux University Hospital (CHU), Cardio-Thoracic Unit, F-33600 Bordeaux, France; 2 IHU Liryc, Electrophysiology and Heart Modeling Institute, Fondation Bordeaux Université, Université de Bordeaux, F-33600 Bordeaux, France; 3 Department of Cardiology, University Hospital Clermont-Ferrand, 63000 Clermont-Ferrand, France; 4 Department of Cardiology, University Hospital Rangueil, 31400 Toulouse, France; 5 Division of Cardiology, University of Ottawa Heart Institute, Ottawa, ON K1Y 4W7, Canada

**Keywords:** implantable cardioverter–defibrillator, remote monitoring, T-wave, oversensing, algorithm

## Abstract

Background: Implantable cardioverter–defibrillators (ICDs) need to reliably detect ventricular tachycardia (VT) and ventricular fibrillation (VF) while avoiding T-wave oversensing (TWOS), which is associated with a risk of inappropriate therapies. The incidence of TWOS with endovascular ICDs appears to differ between manufacturers. Objectives: We aimed to evaluate the incidence and clinical consequences of TWOS with contemporary Medtronic and Boston Scientific ICDs. Methods: Consecutive patients implanted with a recent Medtronic or Boston Scientific ICD and remotely monitored at three French centers were included. All transmitted EGMs labelled as VF, VT, non-sustained VT (NSVT), or ventricular oversensing (Medtronic) were screened for TWOS. Results: Among 7589 transmitted episodes from 674 patients with a Boston Scientific ICD, we did not identify a single case of TWOS. Among 16,790 transmitted episodes from 1733 patients with a Medtronic ICD, we identified 60 patients (3.4%) with at least one episode of TWOS. In 46 patients, TWOS was intermittent (NSVT episodes). In the remaining 14 patients, TWOS resulted in 60 sustained episodes (completed counters). No inappropriate therapies were delivered in 12 of these patients because no therapies were programmed (in monitor zones, 11 episodes) or because therapies were inhibited by the morphology discriminator (Wavelet, 19 episodes) or by the anti-TWOS algorithm (26 episodes). Two patients received inappropriate therapies due to TWOS (0.1% of patients with Medtronic ICDs). Conclusion: On review of 24,379 transmitted episodes from 2407 patients with endovascular ICDs, we found no case of TWOS with Boston Scientific devices, whereas TWOS was not uncommon with Medtronic devices. However, the risk of inappropriate therapy with Medtronic ICDs was very low (0.1%) due to the often intermittent nature of this phenomenon, the morphology discriminator, and the anti-TWOS algorithm.

## 1. Introduction

To properly detect ventricular arrhythmias, implantable cardioverter–defibrillators (ICDs) need to reliably sense R-waves while not sensing other events such as T-waves during sinus rhythm. T-wave oversensing (TWOS) occurs when the T-wave is sensed as an additional ventricular event, effectively doubling the actual ventricular rate as there are two device-defined RR intervals, the R–T and T–R interval, for each true physiological R–R interval. TWOS in pacemaker-dependent patients may cause pacing slower than the lower rate limit [1,2]. In cardiac resynchronization therapy, TWOS may also present as loss of biventricular pacing [3,4]. Most importantly, in patients with ICDs, TWOS may cause inappropriate therapy delivery during sinus tachycardia when both the R-T and T-R intervals surpass tachycardia detection rates [5,6]. Contemporary ICDs employ dynamic proprietary sensing algorithms to detect the low-amplitude, high-frequency signals that occur during ventricular fibrillation (VF) and ventricular tachycardia (VT) while concomitantly minimizing the likelihood of TWOS. In the past, TWOS was reported in up to 14% of patients with ICDs and was a major source of inappropriate therapies. However, a more recent meta-analysis reported an inappropriate therapy rate from TWOS of 0.7% [7]. Differences in filters and hardware among ICD manufacturers may mean TWOS is a manufacturer-dependent issue [8,9,10]. In our device clinic (with over 8000 patients under remote monitoring), we noted that while the occurrence of TWOS was extremely rare with Boston Scientific devices, it appeared to be relatively frequent with Medtronic devices. Medtronic designed a specific algorithm to identify TWOS and withhold inappropriate therapy without requiring operator intervention [11]. Nevertheless, the incidence and clinical importance of TWOS with contemporary Medtronic and Boston Scientific ICDs is not known. 

In the present study, we retrospectively evaluated the incidence and clinical consequences of TWOS with newer-generation ICDs from Medtronic and Boston Scientific.

## 2. Methods

Consecutive patients implanted with a recent Medtronic (Evera, Visia, Claria, or Cobalt) or Boston Scientific (Autogen, Incepta, Resonate, or Momentum, St. Paul, MN, USA) single-, dual-, or triple-chamber ICD and followed using remote monitoring on Carelink or Latitude platforms at one of three French centers were included. All transmitted EGMs labelled by the ICD as non-sustained VT (nsVT), VT (completion of the counter), VF, and ventricular oversensing for Medtronic devices were screened for occurrence of at least one cycle of TWOS by an electrophysiologist (PB) experienced with ICDs and EGM interpretation [12,13,14]. The research reported in this paper adhered to the Helsinki Declaration, data were de-identified prior to analysis, and the ethics review board of Bordeaux University approved the study under reference CER-BDX 2023–46.

### 2.1. Sensing of Ventricular Events

Before comparing TWOS behavior between Boston Scientific and Medtronic devices, it is important to have a basic notion of how their ICDs sense ventricular events. Both companies use adaptive sensing, meaning that the ICD becomes more and more sensitive during the cardiac cycle. This is now standard in all ICDs as it prevents R-wave double counting and also TWOS. In Boston Scientific ICDs, after sensing a ventricular event, there is a refractory period of 135 ms. The sensitivity then decreases to 75% of the sensed peak (or, if the last ventricular event is paced, to 75% of the average peak). Importantly, full R-wave range is available up to 32 mV. The sensitivity setting is then decreased every 35 ms to 7/8 of the previous setting. In Medtronic ICDs, after a ventricular sensed event and a blanking period of 120 ms, the sensitivity also starts at 75% of the peak EGM amplitude, similar to Boston Scientific ICDs. However, the sensitivity value is capped at a maximum of eight times the programmed value (in most cases, meaning 8 × 0.3 mV = 2.4 mV). This makes the ICD more sensitive in the beginning of the cycle and may partially explain why Medtronic ICDs are more prone to TWOS. In addition, visual inspection of ventricular signals between the companies raises the impression that there are differences in filtering, but this information is not public.

### 2.2. TWOS Rejection Algorithm: Basis of Operation

Boston Scientific does not have a dedicated algorithm concerning TWOS. The technical functioning of the Medtronic TWOS rejection algorithm has been extensively described [11].This proprietary algorithm is designed to withhold therapies when the device recognizes TWOS without interfering with the detection of true ventricular arrhythmias (no change in the sensing circuitry). By default, the algorithm is switched ON. This algorithm is based on the recognition of the physical differences between the R- and T-waves and on the alternation of these 2 signals. The slew rate of the R-wave is generally higher than that of the T-wave, a difference that can be amplified by a high-pass differential filter. The algorithm examines differences in amplitude, slew rate, and pattern to distinguish R from T. The analysis becomes active once a rapid ventricular rate is detected (2 consecutive beats for the VT counter, 3 beats for the VF counter). Only ventricular-sensed events above the programmed VT or VF detection zones are analyzed (it does not operate in the VT monitor zone). This algorithm applies to both initial detection and to redetection without limitations imposed by the supraventricular tachycardia limit or the high-rate time out. It distinguishes between the R- and T-waves based on 4 steps: (1) discriminating R-waves from T-waves through differential filtering of the sensing signal; (2) determining the R- and T-wave discrimination thresholds; (3) re-evaluating R- and T-waves; and (4) applying the discrimination criteria to R- and T-waves. Additional criteria include the following: the amplitude of the tentative R- and T-waves must be stable on filtered EGM, the R-T coupling interval must be stable, the R/T ratio from the filtered EGM must be smaller than the R/T ratio from the differential EGM, and the R-T pairs must satisfy a pattern of alternating R- and T-waves. The corrected R-R interval is calculated. If all criteria are satisfied in the set of 6 intervals, it is classified as TWOS and a counter is incremented by 1. If any criterion is not met, the set is classified as normal (no TWOS). The next group of 6 events is then evaluated, using a rolling window. As long as 4 of the last 20 cycles satisfy the T-wave criteria, TWOS is confirmed.

### 2.3. Statistics

Continuous variables are reported as mean ± standard deviation. Implant (baseline) and follow-up parameters were compared using paired *t* tests with *p* < 0.05 considered indicative of statistical significance. All analyses were performed using SPSS 12.0 (SPSS Inc., Chicago, IL, USA). 

### 2.4. Data Availability

The datasets generated and analyzed during the current study are available from the corresponding author on reasonable request.

## 3. Results

**Figure 1:**
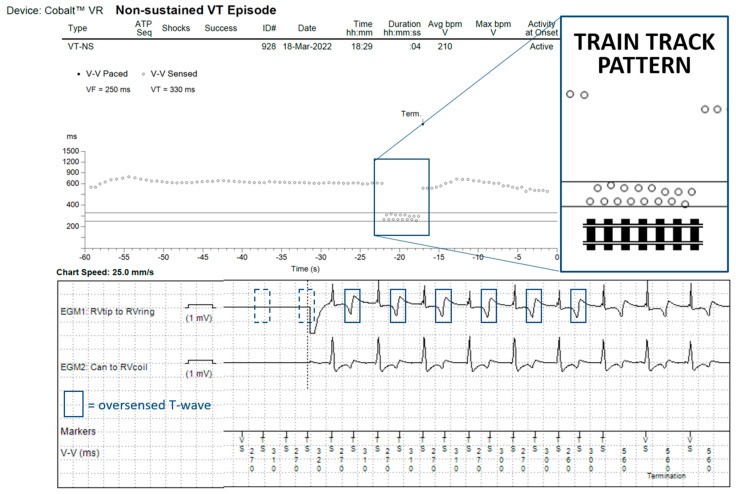
Episode recorded as nsVT showing intermittent TWOS (blue squares) resulting in a “train track” pattern as the alternating R-T and T-R intervals form two separate lines. The TWOS self-terminates before detection of VT/VF is reached.

We included 674 patients with a Boston Scientific ICD, who transmitted a total of 7589 nsVT/VT/VF episodes, and 1733 patients with a Medtronic ICD, who transmitted a total of 16,790 episodes. On review of all episodes transmitted by a Boston Scientific ICD, we did not identify any case of TWOS. Within the episodes transmitted by a Medtronic ICD, we identified 60 patients (3.4%) with at least one episode of TWOS (*p* < 0.001 for the comparison between ICD manufacturers). In 46 of these patients (77%), TWOS was intermittent and resulted in the registration of one or multiple nsVTs (example in Figure 1), never meeting detection criteria.

**Figure 2:**
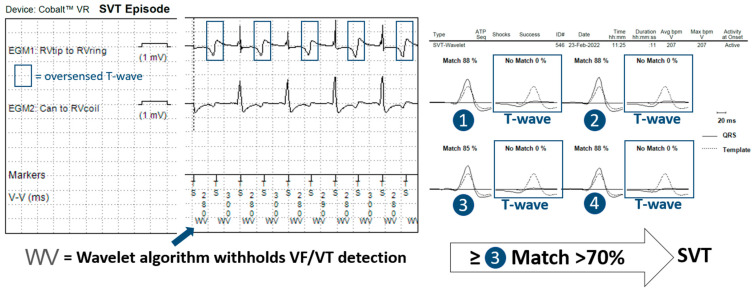
Episode recorded as SVT showing T-wave oversensing (blue squares) resulting in an alternating match/no match pattern. Given that at least 3 complexes match with the template, the Wavelet algorithm inhibits inappropriate therapies.

**Figure 3:**
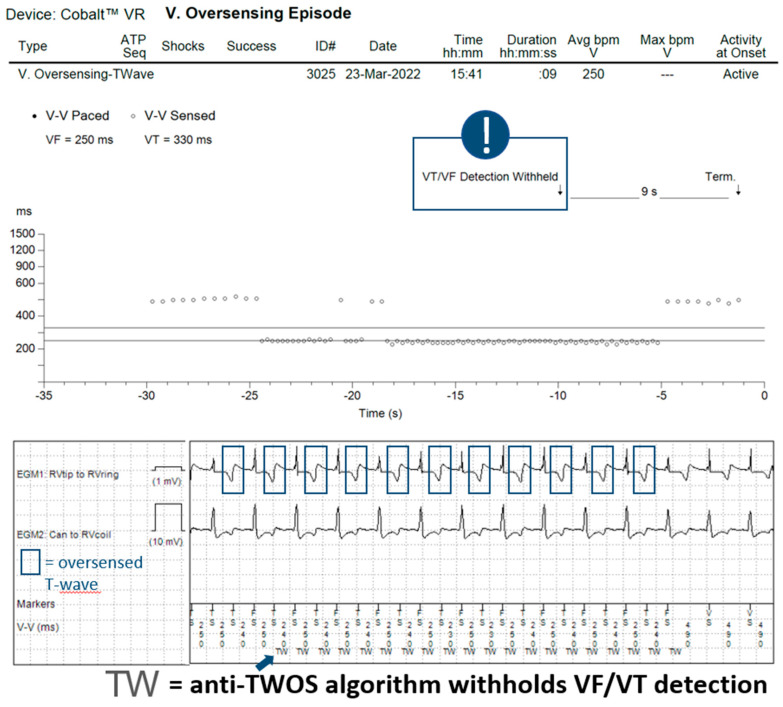
Episode recorded as ventricular oversensing showing TWOS (blue squares) correctly detected by the anti-TWOS algorithm. The “TW” markers (blue arrow) indicate that the anti-TWOS algorithm is active and inhibits VF/VT therapies.

**Figure 4:**
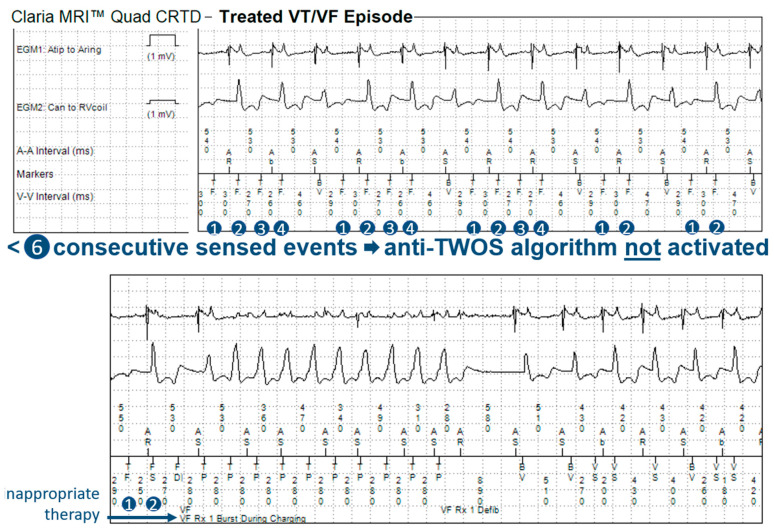
Episode recorded as treated VF/VT showing TWOS not detected by the anti-TWOS algorithm because the criterion requiring 6 consecutive sensed events is not met and because of the presence of intermittent ventricular pacing.

**Figure 5:**
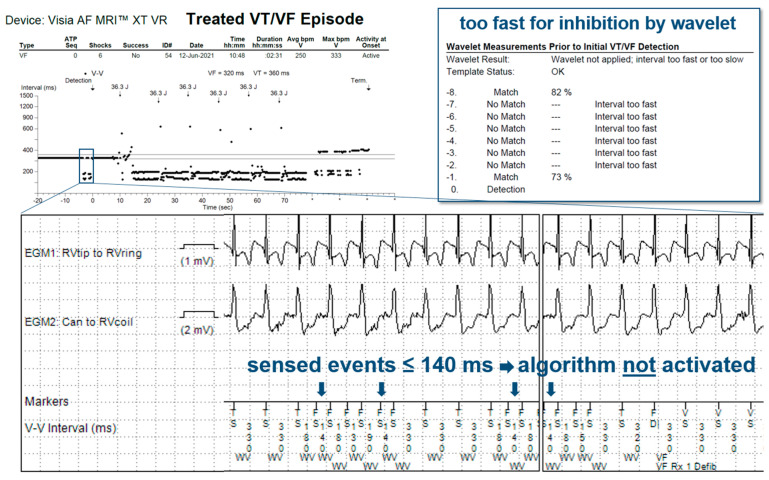
Episode recorded as treated VF/VT showing TWOS not detected by the anti-TWOS algorithm as is it disabled by intervals ≤140 ms between sensed events (blue arrows). The Wavelet algorithm is not applied as the rhythm is faster than the SVT V limit (260 ms).

In 14 patients (23%), the tachycardia detection criteria were met in a total of 60 episodes. In 11 of these episodes, the anti-TWOS algorithm did not intervene, but no therapy was delivered as they fell within a VT monitor zone. In 19 episodes, the Wavelet (morphology) algorithm inhibited wrongful detection of the VT/VF episode, classifying it as SVT without intervention of the anti-TWOS algorithm (Figure 2). In the remaining 30 episodes, the detected heart rate surpassed the SVT-limit, and Wavelet was not interrogated. The anti-TWOS algorithm correctly inhibited therapies in 26 (example in Figure 3) of the 30 episodes (87%). In three patients, the anti-TWOS algorithm failed, resulting in two episodes of the capacitor charging but ultimately no shock being delivered, one episode of ATP delivery (Figure 4), and one episode of six shocks being delivered (Figure 5). Therefore, overall, two patients (0.1%) had inappropriate therapies due to TWOS. 

In 26 patients (43%) with TWOS, no programming changes were performed as the over-sensing of the T-wave was only responsible for short nsVT episodes. None of these patients had an inappropriate therapy during follow-up. In the remaining 34 patients (57%), one or multiple programming changes were performed with either a change in the sensing polarity in 21 patients (true bipolar to integrated bipolar), a change in the ventricular sensitivity from 0.3 mV to 0.45 or 0.6 mV in 15 patients, or a change in detection zones in 18 patients. In the patient with an aborted capacitor charge and the patient with an aborted charge and inappropriate ATP, reprogramming of the sensitivity and VT detection zones successfully prevented further inappropriate therapies during follow-up. In the third patient with inappropriate shocks, reprogramming the sensing vector to integrated bipolar and modifying the detection zones prevented further inappropriate therapies.

## 4. Discussion 

To better understand the incidence of TWOS with contemporary ICDs and its clinical implications, we performed a retrospective study in a broad cohort of patients with recently implanted ICDs from two leading manufacturers using remote monitoring EGM analysis. The principal finding of our analysis is that the incidence of TWOS varied significantly between manufacturers: TWOS was seen in 3.4% of patients with Medtronic ICDs compared to 0% with Boston Scientific ICDs. However, despite the higher incidence of TWOS seen with Medtronic ICDs, our data indicate that it rarely results in inappropriate therapies (0.1%). 

A fundamental requirement for ICD systems is reliably sensing low-amplitude and variable signals during VF without oversensing T-waves during sinus/supraventricular rhythms. Differences in proprietary high-pass filtering, dynamic adjustment of sensing thresholds, and sensing bipoles may explain the differences observed in the incidence of TWOS between Medtronic and Boston Scientific devices. Differences in sensing hardware may at least in part account for the difference, particularly when the R-wave is of large amplitude. Although dynamic sensitivity adjustments are set at 75% of the R-wave for both manufacturers, it is limited to 8 times the programmed sensitivity for Medtronic (0.3 mV by default). For example, when an R-wave measures 20 mV, the sensitivity is adjusted to 2.4 mV with Medtronic, whereas it is adjusted to 15 mV with Boston Scientific. The risk of oversensing a T-wave would be expected to considerably differ accordingly. This difference in automatic sensitivity adjustment would not explain the lack of TWOS seen with Boston Scientific ICDs when the R-wave is of low amplitude, however. Details of the proprietary filter settings of each manufacturer are not readily available, but it appears that differences in bandwidth filters may contribute to the discrepancy in the incidence of TWOS with their ICDs. Optimizing devices’ diagnostic specificity by reducing the risk of oversensing must not come at the price of reduced sensitivity given the potentially fatal consequences of undersensing a ventricular arrhythmia. While it is possible to quantify the incidence of TWOS, it is more difficult, if not impossible, to quantify the incidence of undersensing of ventricular arrhythmias as such episodes may not be recorded. The potential for an increased risk of ventricular undersensing with Boston Scientific devices due to more aggressive filters therefore remains theoretical and cannot be addressed in our study.

When analyzing the incidence of TWOS, it is important to differentiate between the number of patients with at least one episode of TWOS, the number of patients with a sustained episode that is detected as a sustained tachyarrhythmia, and the number of patients who receive inappropriate therapies as a result. In our sample, few patients with sustained episodes of TWOS received ICD therapies due to episodes falling within VT monitor zones where no treatments are programmed and due to two other algorithms that prevented inappropriate treatments, with perhaps surprising effectiveness. The first algorithm is the morphology discriminator Wavelet, whose principal function is to discriminate between VT and SVT. Although arguably not specifically designed to address TWOS, this algorithm prevents inappropriate therapies by classifying sustained episodes of TWOS as SVT. During an episode of TWOS, half of the detected complexes correspond to the R-wave and therefore match the reference morphology, whereas the other half correspond to the T-wave and therefore do not match. For Wavelet to diagnose SVT, three of eight complexes must match the reference morphology, thus rendering it an effective first line for preventing inappropriate treatments during sustained episodes of TWOS during which four of eight complexes are expected to match. Similarly, for Boston Scientific ICDs, Rhythm ID behaves in a similar way, rejecting ventricular arrhythmia when at least 3 out of 10 complexes match (during TWOS, 5 out of 10 complexes are expected to match). However, our data show that the effectiveness of the Medtronic algorithm is incomplete. Wavelet is not applied in certain scenarios such as when the interval between sensed events is equal to or shorter than the SVT limit (260 ms by default) or when the PR logic discriminator declares VT (when V > A). The number of sustained episodes of TWOS in our study that were not intercepted by the morphology discriminator was modest: 30 episodes in total. When this first algorithm fails to inhibit therapies, a second algorithm designed specifically for this function is used with relatively strict criteria that must be met so as to not compromise sensitivity for VF detection. We report high accuracy for this anti-TWOS algorithm, but it too is not perfect. All episodes classified as TWOS by the algorithm were correct. However, in 13% of cases, the anti-TWOS algorithm failed to diagnose TWOS and therefore failed to prevent inappropriate ICD therapies. This algorithm may fail in a few scenarios, including consistent or intermittent ventricular pacing, short “RR” intervals (<140 ms), and very low or very large R-wave amplitudes (filtered and rectified R-wave > 30 mV). 

As this was a retrospective multicenter study, there was no standardized management when TWOS was diagnosed. This is a major limitation of this study and prevents definitive conclusions being drawn in terms of how best to manage patients in whom TWOS is identified. In our sample, programming modifications were most often reactionary and performed when a sustained episode occurred. Thus, in most patients, no modifications were performed as the episodes were not sustained. The underlying mechanism causing TWOS determined the intervention chosen. When the R-wave was of large amplitude, TWOS was typically managed by raising the sensitivity threshold, adjusting the tachycardia zones, and/or increasing the detection interval counter. The effectiveness of these programming interventions was not usually assessed in real time since TWOS was intermittent. In patients with relatively low-amplitude R-waves, there is a specific programming option only available in Medtronic devices, which is to change the RV lead’s sensing configuration from *true* to *integrated* bipolar. This noninvasive method can resolve TWOS but may result in P-wave or diaphragmatic oversensing in certain patients. Switching from bipolar to integrated bipolar sensing can increase the R-wave amplitude but may not always rectify the issue of TWOS. When noninvasive efforts to eliminate TWOS fail, invasive measures may be required, including ventricular lead revision. Another potential solution, which is supported by the results of this study, could be to replace the pulse generator with a Boston Scientific device [15]. 

## 5. Study Limitations

The most important limitation of this study is its retrospective nature and the variable management strategies implemented in response to diagnosed TWOS as mentioned above. We also could not investigate a probable association between underlying cardiac and the occurrence of TWOS. All EGM data were based on remote monitoring transmissions; therefore, episodes of TWOS may have been missed. However, established dedicated protocols to avoid missed transmissions at our remote monitoring centers and the large number of included episodes (>24,000) limit the risk of this bias. We only analyzed the results for two companies. However, we specifically chose Boston Scientific and Medtronic because, in our experience, these are the two manufacturers where we felt the differences between TWOS were the greatest.

## 6. Conclusions

In our analysis of 24,379 transmitted episodes from 2407 patients with contemporary endovascular ICDs, we identified a significantly higher incidence of TWOS with Medtronic ICDs than with Boston Scientific ICDs (3.4% vs. 0%). However, the risk of inappropriate therapy was very low (0.1%) thanks to the often intermittent nature of TWOS, the morphology discriminator, and the anti-TWOS algorithm.

## Data Availability

Data will be made available upon reasonable request.
